# Herbal medicine Shaofu Zhuyu decoction for primary dysmenorrhea: a systematic review protocol

**DOI:** 10.1186/s13643-016-0185-9

**Published:** 2016-01-20

**Authors:** Hoyoung Lee, Tae-Young Choi, Chang-Seon Myung, Myeong Soo Lee

**Affiliations:** KM Fundamental Research Division, Korea Institute of Oriental Medicine, 483 Expo-ro, Yuseong-gu, Daejeon 305-811 Republic of Korea; Clinical Research Division, Korea Institute of Oriental Medicine, 483 Expo-ro, Yuseong-gu, Daejeon 305-811 Republic of Korea; Department of Pharmacology, Chungnam National University College of Pharmacy, 99 Daehakno, Yuseong-gu, Daejeon 305-764 Republic of Korea

**Keywords:** Herbal medicine, Shaofu Zhuyu decoction, Dysmenorrhea, Randomized controlled trials (RCTs), Systematic review

## Abstract

**Background:**

Dysmenorrhea is a common gynecological complaint in adolescent and young females. The purpose of this study is to assess the efficacy of Shaofu Zhuyu (SFZY) decoctions as treatments for primary dysmenorrhea.

**Methods/design:**

Fifteen (four English, seven Korean, three Chinese, and one Japanese) databases will be searched from their inception without a language restriction. These include PubMed, AMED, EMBASE, The Cochrane Library, seven Korean Medical Databases (Korean Studies Information, DBPIA, Oriental Medicine Advanced Searching Integrated System, Research Information Service System, KoreaMed, The Town Society of Science Technology, and the Korean National Assembly Library), three Chinese Medical Databases [the Chinese Medical Database (CNKI), Chongqing VIP Chinese Science and Technology Periodical Database (VIP), and WanFang Database], and one Japanese Database (J global). Randomized clinical trials (RCTs) included those that examined an SFZY decoction or a modified SFZY decoction. The control groups include no treatment, placebo, and medication. Trials testing a combination of SFZY decoction and medication compare to the same medication alone will be also included. Data extraction and risk of bias assessments will be performed by two independent reviewers. All statistical analyses will be conducted using Review Manager software (RevMan V.5.3.0). Methodological quality will be assessed with the Cochrane risk of bias tool.

**Discussion:**

This systematic review will provide a detailed summary of the available evidence testing the effects of SFZY decoctions for the treatment of primary dysmenorrhea. The review will benefit patients and practitioners in the fields of traditional and complementary medicine.

**Systematic review registration:**

PROSPERO registration number: CRD42015016386

## Background

### Description of the condition

Dysmenorrhea is a common gynecological complaint in adolescent and young females. Dysmenorrhea is characterized by lower abdominal pain that occurs during menstruation [1]. Different types of studies have found a consistently high prevalence of dysmenorrhea in women of different ages and nationalities with an estimated prevalence ranging from 45 to 97 % [2]. Ten percent of these women suffer from symptoms severe enough to render them incapacitated for 1 to 3 days each menstrual cycle [3]. Period pain can lead to absences from school or work [4]. In the USA alone, it was estimated that in the mid-1980s, 600 million hours were lost from work, which led to an economic loss of two billion dollars; in today’s dollars, this figure would be much higher [5].

### How the intervention might work

The mechanisms of primary dysmenorrhea have been attributed to high serum levels of prostaglandin E2 (PGE2), prostaglandin F2-α (PGF2-α), and leukotriene [6]. Severe myometrial contractions, vasoconstriction, uterine ischemia, and subsequent dysmenorrheic pain result from the release of these cytokines. Moreover, withdrawal of progesterone before the beginning of the menstrual cycle initiates arachidonic acid release and further elevates cytokine levels due to the degradation of arachidonic acid. Higher cytokine levels contribute to a higher intensity of dysmenorrheic pain and associated symptoms [7]. Non-steroid anti-inflammatory drugs (NSAIDs), therefore, are the primary treatment for this condition but are limited by inadequate pain control, gastrointestinal discomfort, and an impact on renal function. Combined oral contraceptives are also frequently used but are not universally accepted possibly due to their potential side effects including inducing endometriosis [8]. Therefore, complementary and alternative medicine (CAM) is in high demand in many countries [9]. In one large study, as many as 48 % of women reported the use of CAM as an alternative to prescription medication or to enhance the effectiveness of their prescription medications [10, 11]. Recenlty, it was reported that Shaofu Zhuyu (SFZY) decoction have the efficacy of uterine smooth muscle constriction and manifested an anti-inflammatory efficacy [12]. Also, SFZY decoction improved hemorheological factor of blood stasis and regulation for activity on rat ovary [13].

### Description of the intervention

Herbal medicine is currently used in hospitals and clinics in Korea [14], China [2], Taiwan [15], and Japan [16] for the treatment of primary dysmenorrhea. SFZY decoction was first described in the *Yi Lin Gai Cuo*, which is a famous formula that has been used for treating primary dysmenorrhea in China since the Qing dynasty. This decoction is used, particularly in gynecology, for blood stasis accompanied by masses and gatherings in the lower abdomen [17]. Clinically, it has been used for the treatment of chronic pelvic inflammatory disease, infertility, endometrial hyperplasia, myoma uteri, and uterine cancer [18]. Many reports have described its efficacy for treating vascular disorders and pain [19], endometriosis [20], cancer [21], and menstrual irregularities in vivo [22]. SFZY decoction composed of ten herbs by Quin-ren Wang in Qing dynasty: Fructus Foeniculi, Zingiberis Rhizoma, Cinnamomi Cortex, Paeoniae Rubra Radix, Angelica Sinensis Radix, Carthami Flos, Myrrha, Corydalis Rhizoma, Typhae Pollen, and Trogopterori Faeces, in the ratio of 0.5:1:1:1:3:1:1:1:3:2 on a dry weight basis [12]. The detail of composition is shown Table 1. The composition of China came from Qing dynasty and decoction of Korean came from DongUiBoGam. SFZY decoctions have shown an effect on uterine muscles and may help to prevent and cure dysmenorrhea. SFZY decoctions are considered an effective prescription for treating primary dysmenorrhea [23], which has been reported as one of the most common gynecological disorders in young women [24].

**Table 1:** Compositions of Shaofu Zhuyu decoction

Name of herbs	Scientific name	Amount (g)		
		China^a^	Modify^b^	Korea^c^
Foeniculi Fructus	*Foeniculum vulgare Mill.*	0.5	0.5	4.0
Zingiberis Rhizoma	*Zingiber officinale Roscoe*	1.0	1	0.8
Carthami Flos	*Corydalis ternata Nakai*	1.0	2	4.0
Myrrha	*Commiphora molmol Engler*	1.0	1	4.0
Angelicae Sinens Radix	*Angelica gigas N.*	3.0	3	12.0
Cnidii Rhizoma	*Cnidium officinale Makino*	1.0	1	4.0
Cinnammomi Cortex	*Cinnamomum loureirii Nees*	1.0	1	4.0
Paeoniae Rubra Radix	*Paeonia obovata Maxim*	1.0	2	8.0
Typhae Pollen	*Typha angustifolia L.*	3.0	1	12.0
Trogopterori Faeces	*Trogopterus xanthipes*	2.0	3	8.0

^a^Qing-ren Wang, “Correction of Errors in Medical Classics”

^b^Xiaochen Huang et al., J Chromatogr B Analyt Technol Biomed Life Sci. 2014; 1;962:75–81

^c^Jun Heo, “DongUiBoGam”

### Why is performing this review important?

Recently, many studies have reported treatments using herbal formulas. The Cochrane Review on Chinese herbal medicine showed promising evidence for the use of Traditional Chinese Medicine (TCM) in reducing menstrual pain in primary dysmenorrhea. Unfortunately, many of the studies supporting the use of Chinese herbs were of poor quality [25, 26].

Currently, no relevant systematic reviews of the efficacy of SFZY decoctions for treating primary dysmenorrhea are conducted.

### Objectives

The aim of this study is to systematically review the available literature regarding the efficacy of SFZY decoctions in treating primary dysmenorrhea.

## Methods/design

This study has been registered with international Prospective Register of Systematic Reviews (PROSPERO): CRD42015016386.

### Criteria for including studies in this review

#### Type of studies

Only randomized controlled trials (RCTs) and quasi-RCTs will be included.

#### Type of participants

This study will include women of reproductive age with primary dysmenorrhea, i.e., individuals with no identifiable pelvic pathology as indicated by a pelvic examination, ultrasound scans and laparoscopy, or women self-reporting a diagnosis of primary dysmenorrhea.

#### Types of interventions

Studies that used an SFZY decoction or a modified SFZY decoction will be included. SFZY decoctions will include the following ten formulas: Fructus Foeniculi, Zingiberis Rhizoma, Cinnamomi Cortex, Paeoniae Rubra Radix, Angelica Sinensis Radix, Carthami Flos, Myrrha, Corydalis Rhizoma, Typhae Pollen, and Trogopterori Faeces [17]. Modified SFZY decoction formulas will be included as well. Modified SFZY decoctions prescribe according to TCM syndrome differentiation will be acceptable and be defined by practitioners as adding only herbs to the original herbs, resulting in nearly the same actions as the original SFZY decoction. All types of herbal medicines will be included. There is no limitation on the number of herbs, administration methods dosage, or duration of treatment.

#### Types of comparisons

The control groups will consist of no treatment, placebo, and medication. Trials examining a combination of SFZY decoctions and medication will compare to the same medication alone will be also included.

### Outcome measures

#### Primary outcomes

1. Pain: a reduction in pain (i.e., menstrual pain) that occurs only during the intervention or occurred as a result of the intervention, measure by a visual analogue scale (VAS), other validated scales, or as a dichotomous outcome.
2. Response rate: an overall reduction in symptoms (other menstruation-related symptoms) that occurs only during the intervention or occurred as a result of the intervention, measure by changes in dysmenorrhea symptoms and treatment effectiveness, and is either self-reported, observed, or reported by other similar measures.

#### Secondary outcomes

1. Adverse effects: measured by any relevant incidence and duration of any side effects.
2. Quality of life: measured by a validated scale.

### Search methods for identifying the studies

#### Electronic searches

The following databases will be searched from their inception: PubMed, AMED, EMBASE, The Cochrane Library, seven Korean Medical Databases (Korean Studies Information, DBPIA, Oriental Medicine Advanced Searching Integrated System, Research Information Service System, KoreaMed, The Town Society of Science Technology, and the Korean National Assembly Library), three Chinese Medical Databases [the Chinese Medical Database (CNKI), Chongqing VIP Chinese Science and Technology Periodical Database (VIP), and WanFang Database], and one Japanese Database (J global).

#### Other sources

Studies will also be obtained from the following sources:

- The reference lists of all relevant articles
- Hand searching of department files
- Unpublished conference proceedings relevant to primary dysmenorrhoea will be reviewed, if available

#### Search strategy

The strategy for searching the databases is presented in Tables 2 and 3. Similar search strategies will be applied for all databases. In addition, the reference lists of all retrieved articles will be hand-searched for further relevant literature. Hard copies of all included articles will be read in full. Because all of the various databases use for this study possessed their own subject headings, each database will be searched independently.

**Table 2:** Search strategy used in PubMed

Number	Search items
1 Related to intervention	少腹逐瘀汤
2	Shaofu Zhuyu decoction
3	Shaofu Zhuyu formula
4	Shaofu Zhuyu tang
5	1 or 2–5
6 Related to disease	Dysmenorrhea
7	Menstruation disturbances
8	Menstrual disorder
9	Pelvic pain
10	Painful menstruation
11	Painful period
12	Period pain
13	Primary dysmenorrhea
14	7 or 8–14
15 Related to study design	Randomized controlled trial
16	Controlled clinical trial
17	Randomized
18	Placebo
19	Drug therapy
20	Randomly
21	Trial
22	Groups
23	#15 OR #16 OR #17 OR #18 OR #19 OR #20 OR #21 OR #22
24	Animals NOT humans
25	#23 NOT #25

Any words containing this searching item will be searched. This search strategy will be suitable for other electronic databases

**Table 3:** Search strategy used in CNKI

Number	Search items
1 Related to intervention	少腹逐瘀汤
2	Shaofu Zhuyu decoction
3	Shaofu Zhuyu formula
4	Shaofu Zhuyu tang
5	1 or 2–5
6 Related to disease	痛经
7	原发性痛经
8	月经痛
9	经期腹痛
10	经痛
11	Dysmenorrhea
12	Primary dysmenorrhea
13	Menstrual disorder
14	Pelvic pain
15	Menstruation disturbances
16	6 or 7–15
17 Related to study design	随机
18	对照
19	临床研究
20	Controlled trial
21	Randomized controlled trial
22	17 or 18–22
23	6 and 16 and 22

### Data collection and analysis

#### Selection of studies

**Fig. 1:**
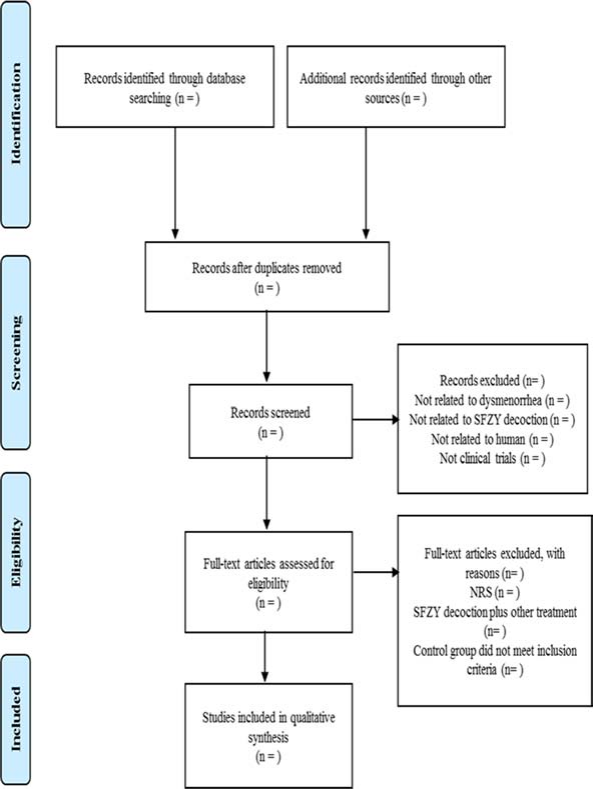
PRISMA diagram for the included studies. *NRS* non-randomized studies, *SFZY* Shaofu Zhuyu

Two reviewers (HYL and TYC) will review and screen the titles and abstracts to identify eligible trials according to the inclusion criteria. Disagreements will be resolved by discussion, if necessary, by the arbiter (MSL). Details of the study selection procedure are shown in Fig. 1.

#### Data extraction

All articles will be performed by two authors (HYL and TYC) who extract data according to pre-defined criteria. Information such as the participants, interventions, outcomes, and results will be obtained from each report. Any disagreement between the two authors will be resolved by discussion. Another author (MSL) will act as an arbiter for unresolved disagreements.

### Assessment of bias in the included studies

Two authors (HYL and TYC) will assess the risk of bias using the following seven criteria from the Cochrane classification: (1) random sequence generation, (2) allocation concealment, (3) blinding of participants and personnel, (4) blinding of outcome assessment, (5) incomplete outcome data, (6) selective outcome reporting, and (7) other sources of bias (we evaluate baseline imbalance) [27]. This review uses “L, U and H” as judgments keys; “Low” indicates a low risk of bias (L), “Unclear” indicates that the risk of bias is uncertain (U), and “High” indicates a high risk of bias (H). Disagreements will be resolved by discussions between all reviewers.

### Data synthesis

All statistical analyses will be conducted using the Cochrane Collaboration’s software program, Review Manager (RevMan), Version 5.3.0 for Windows (Copenhagen, The Nordic Cochrane Center). Differences between the intervention and control groups will be assessed. For the continuous data, we will use the mean difference (MD) with 95 % confidence intervals (CI) to measure the treatment effect. We will convert other forms of data into MDs. In the case of outcome variables with different scales, we will use the standardized mean difference (SMD) with 95 % Cis. For dichotomous data, we will present the treatment effects as a relative risk (RR) with 95 % Cis. We will convert other binary data into the RR form. For studies with insufficient information, we will contact the primary authors to acquire and verify data when possible. The chi-square test for heterogeneity and the *I*^2^ test will be used to evaluate the heterogeneity of the included studies. Unless excessive statistical heterogeneity is present, we will pool the data across studies for the meta-analysis using a fixed effects model.

#### Unit of analysis issues

For cross-over trials, data from the first treatment period will be used. For trials in which more than one control group will be assessed, the primary analysis will combine the data from each control group. Subgroup analyses of the control groups will also be performed. Each patient will be counted only once in the analysis.

#### Dealing with the missing data

Intention-to-treat analyses that include all of the randomized patients will be performed. For patients with missing outcome data, a carry-forward of the last observed response will be used. The individual patient data will be sought from the original source or the published trial reports when the individual patient data are unavailable.

#### Assessment of heterogeneity

We will use the random effects or fixed effects model for the meta-analysis according to the data analysis. If a meta-analysis is possible, we will use the *I*^2^ statistic to quantify the inconsistencies among the included studies. According to the guidance given in *the Cochrane Handbook* for Systematic Reviews of Interventions, as a general rule, *I*^2^ values of up to 25 % provide evidence of low heterogeneity; a value of 50 % is considered moderate heterogeneity and 75 % or above is considered as a high heterogeneity. In the presence of significant heterogeneity, the causes of heterogeneity will be examined by pre-specified subgroup analysis and also sensitivity analysis, if possible. Where subgroup analysis fails to explain the heterogeneity, then data will be analyzed using the random effects model. If heterogeneity is observed, we will conduct a subgroup analysis to explore the possible causes [28].

#### Assessment of reporting biases

If a sufficient number of included studies (at least ten trials) are available, we will use funnel plots to detect reporting biases. However, funnel plot asymmetry is not the same as publication bias; therefore, we will attempt to distinguish the possible reasons for the asymmetry, such as small-study effects, poor methodological quality, and true heterogeneity in the included [29, 30].

#### Subgroup analysis and investigation of heterogeneity

If there are an adequate number of studies, we will conduct subgroup analyses to interpret the heterogeneity between the studies, including the following:

1. Type of design: SFZY decoction treatment used alone or as combination therapy with SFZY decoction and conventional therapy
2. Type of intervention: type of herbal medicines (SFZY decoction or modified SFZY decoction)

#### Sensitivity analysis

We will conduct sensitivity analysis to test the robustness of the primary decisions of the review process. The principal decision nodes conclude methodological quality, sample size and the effect of missing data. The meta-analysis will be repeated, and studies of lower quality will be excluded. The result will be compared and discussed according to the results.

### Ethics and dissemination

Ethical approval is not required, given that this protocol is for a systematic review. The findings of this review will be disseminated widely through peer-reviewed publications and conference presentations.

## Discussion

As a primary data collection will not be undertaken, no additional formal ethical assessment or informed consent is required. The systematic review will be published in a peer-reviewed journal and disseminated electronically or in print. Updates of the review will be conducted to inform and guide healthcare practice and policy. In this review, we will collect data on the safety and efficacy of SFZY decoctions for treatment of primary dysmenorrhea. The review will fuel the development of treatment of primary dysmenorrhea patients using traditional and complementary medicine.

## Abbreviations

CAM: complementary and alternative medicine
CI: confidence intervals
NSAIDs: non-steroid anti-inflammatory drugs
PGE2: prostaglandin E2
PGF2-α: prostaglandin F2-α
RCTs: randomized controlled trials
RR: relative risk
SFZY: Shaofu Zhuyu
TCM: Traditional Chinese Medicine
VAS: visual analogue scale
